# Direct Detection of Viral Infections from Swab Samples by Probe‐Gated Silica Nanoparticle‐Based Lateral Flow Assay

**DOI:** 10.1002/open.202300120

**Published:** 2023-10-12

**Authors:** Dilara Buse Durdabak, Soner Dogan, Serap Demir Tekol, Caner Celik, Veli Cengiz Ozalp, Bilge Guvenc Tuna

**Affiliations:** ^1^ Department of Biophysics Faculty of Medicine Yeditepe University Istanbul 34755 Turkey; ^2^ Department of Medical Biology Faculty of Medicine Yeditepe University Istanbul 34755 Turkey; ^3^ Department of Clinical Microbiology University of Health Sciences Kartal Dr. Lutfi Kirdar City Hospital Istanbul 34865 Turkey; ^4^ Department of Emergency Medical Service Memorial Sisli Hospital Istanbul Turkey; ^5^ Department of Medical Biology Faculty of Medicine Atilim University Ankara 06830 Turkey

**Keywords:** detection of viral infection, fluorescence-based LFA, gated-SNPs, lateral flow assay, silica nanoparticles

## Abstract

Point‐of‐care diagnosis is crucial to control the spreading of viral infections. Here, universal‐modifiable probe‐gated silica nanoparticles (SNPs) based lateral flow assay (LFA) is developed in the interest of the rapid and early detection of viral infections. The most superior advantage of the rapid assay is its utility in detecting various sides of the virus directly from the human swab samples and its adaptability to detect various types of viruses. For this purpose, a high concentration of fluorescein and rhodamine B as a reporting material was loaded into SNPs with excellent loading capacity and measured using standard curve, 4.19 μmol ⋅ g^−1^ and 1.23 μmol ⋅ g^−1^, respectively. As a model organism, severe acute respiratory syndrome coronavirus‐2 (CoV‐2) infections were selected by targeting its nonstructural (NSP9, NSP12) and envelope (E) genes as target sites of the virus. We showed that NSP12‐gated SNPs‐based LFA significantly outperformed detection of viral infection in 15 minutes from 0.73 pg ⋅ mL^−1^ synthetic viral solution and with a dilution of 1 : 10^3^ of unprocessed human samples with an increasing test line intensity compared to steady state (n=12). Compared to the RT‐qPCR method, the sensitivity, specificity, and accuracy of NSP12‐gated SNPs were calculated as 100 %, 83 %, and 92 %, respectively. Finally, this modifiable nanoparticle system is a high‐performance sensing technique that could take advantage of upcoming point‐of‐care testing markets for viral infection detections.

## Graphical abstract

Here, universal‐modifiable probe‐gated silica nanoparticles (SNPs) based lateral flow assay (LFA) is developed in the interest of the rapid and early detection of viral infections. The most superior advantage of the rapid assay is its utility in detecting various sides of the virus directly from the human swab samples and its adaptability to detect various types of viruses. The NSP12, NSP9, and E gene targets of CoV‐2 were used as detection targets.

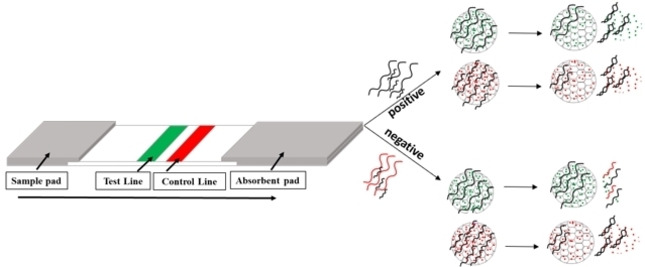

## Introduction

The viruses are small, with different shapes, and most importantly have DNA or RNA genomes, which are responsible for their specific pathogenic behavior. In addition, viruses, specifically RNA viruses, are dangerous types of viruses that could infect humans, animals, and plants and cause deadly diseases.^[1]^ In particular, RNA viruses such as the Zika virus, the Ebola virus, the influenza virus, and the severe acute respiratory syndrome coronavirus 2 (CoV‐2) are potential risk factors for future epidemics since they may cause high mortality rates in highly populated communities.^[2]^ In this context, positive‐stranded RNA viruses are the most dangerous ones since their genomic RNA could be translated immediately into protein when they enter a cell. Considering the CoV‐2 pandemic, which is caused by a positive‐sense RNA virus (CoV‐2), we have experienced the essential need for the rapid detection of viral infection at an early stage of the disease in order to efficiently control the spread of viruses.

The application of nucleic acid‐based detection methods in viral infection diagnosis is accepted to be the gold standard for their reliability since they have high accuracy. However, these methods require long protocols and reaction time, as well as experienced technicians and costly equipment and materials. Moreover, amplification‐based methods are prone to contamination, leading to false positives.[^3^, ^4^] Therefore, a fast, easily applicable, cheap, and rapidly modifiable detection method is needed for the detection of viral agents. In the current study, we developed a fast universal‐modifiable point‐of‐care method for viral infections using CoV‐2 viral RNA as a proof‐of‐concept model.

CoV‐2 is a positive‐sense single‐stranded RNA virus that consists of structural and nonstructural proteins encoded by the Spike (S), Nucleocapsid (N), Envelope (E), and Membrane (M) genes. Nonstructural proteins are encoded by viral polyproteins ORF1a and ORF1b regions. These proteins are involved in genetic processes, replication, transcription, and translation. In this context, the main component of ORF1b is RNA‐dependent RNA polymerase (RdRp) which is responsible for viral RNA synthesis and its central role in the replication and transcriptional cycle of CoV‐2 by nonstructural protein‐12 (NSP12).^[5]^ Furthermore, nonstructural protein‐9 (NSP9) plays a role in the replication complex. The expression of the NSP9 protein is thought to be required for viral replication to take place.^[6]^ Therefore, E, NSP12, and NSP9 sites of the CoV‐2 virus are crucial targets in the fight against CoV‐2 viral infection in practice.

In order to diagnose the CoV‐2 virus using specimens from the upper respiratory tract, and nasopharyngeal swabs, the RT‐qPCR method is used as the gold standard technique. In this process, the range of viral load could vary from 10^3^ to 10^10^ copies ⋅ mL^−1^ in nasal swab samples.^[7]^ In addition, in this method, the detectable viral RNA amplification is graphically referred to as the quantification cycle, often reported as the cycle threshold (Ct) value. For diagnosis purposes, different Ct values ranging from 16.9 to 38.8 have been used for nasal swab samples taken from numerous individuals in clinics. On the other hand, one of the studies has reported that a Ct value bigger than 40 Ct was considered negative.^[8]^ Although RT‐qPCR is a reliable method, the Ct cut‐off values could vary due to the individual subjects, sampling times, and procedures used by the technicians in diagnostic centers. Moreover, using the upper respiratory tract samples, the sensitivity of the RT‐qPCR is reported to be around 82 % in one of the recent studies.^[9]^ They also reported up to a 90 % increase in sensitivity when two consecutive tests were performed for the same individual subject. On the other hand, there are some limitations to performing RT‐qPCR in practice, since the application of consecutive tests may not be practical due to the availability of the test kits in the diagnosis center, the cost of the detection tests, and the time needed for the process. Besides, CoV‐2 viruses may evolve quickly with a variety of mutation types as we are experiencing worldwide currently.^[10]^ For this reason, gold standard detection methods like RT‐qPCR, may not be able to detect every variant of viral infections due to a lack of correct primer availability. In parallel to this, the Centers for Disease Control and Prevention (CDC) and World Health Organization (WHO) urgently support rapid diagnostic methods to keep viral infection under control rapidly.^[11]^ To overcome this issue, many detection methods have been developed for the detection of CoV‐2 in recent years. Here, we are reporting a nanoparticle‐based rapid detection assay for the diagnosis of the CoV‐2 virus to mitigate challenges that may be experienced with the RT‐qPCR method such as detection time, accuracy, simplicity, affordability, suitability for mass production, and importantly universal‐modifiability.

The application of novel engineered materials nanomaterials which could be used for a variety of purposes including in medical applications has increased significantly since the 1980s.^[12]^ In this context, mobile composite material number‐41 (MCM‐41) which is one of the silica‐based nanomaterials is used in targeting therapy and diagnostic studies thanks to its functional high surface area and most importantly uniform controllable porous structure with excellent loading capacity.^[13]^ This important ability gives MCM‐41 the potential to carry a high‐concentration template in every individual usage with a desired amount to sense a specific material like fluorescein,^[14]^ rhodamine^[15]^ with gated molecules including aptamers, oligonucleotides, and antibodies. On the other hand, the potential usage of SNPs has been extended for viral,[^14^, ^16^] bacterial,^[17]^ cancer biomarker,^[18]^ and single nucleotide polymorphism diseases^[19]^ detection in different specimens using gated designs. Although the application of SNPs has become more common in recent years there are some caveats. For example, in one of the studies, aptamer probes‐gated SNP‐based methods were developed for *Staphylococcus aureus* (*S. aureus*) detection with a limit of detection (LOD) 17 CFU/mL.^[20]^ Moreover, the detection of bacterial infections using aptamer probe‐gated SNPs was also reported in samples of food, environmental surfaces, and live organisms.[^21^, ^22^, ^23^, ^24^, ^25^, ^26^] In another study, as a viral infection, CoV‐2 was determined using aptamer‐gated methylene blue@mesoporous silica film/laser engraved graphene electrode as a LOD 0.36 ng/mL.^[27]^ Furthermore, single‐stranded oligonucleotide‐gated systems target various molecules such as small molecules like ATP,^[28]^ mRNA,^[29]^ and genomic DNA.^[19]^ In this context, recently, we reported the potential usage of SNPs in proof‐of‐concept design for the detection of CoV‐2 viral infection using both synthetic DNA and human samples.^[14]^ In here, the fluorescein‐loaded single‐stranded oligonucleotide (probe) gated SNPs were used for CoV‐2 detection via in vitro tube releasing procedure with a LOD 48 fM within 15 mins without any additional amplification steps. However, this concept could not be used in clinical practices directly due to the lack of technical instruments and experienced personnel. Therefore, we aimed to develop a rapid, sensitive detection assay and to make our previous study applicable. The current study is based on the sensing materials loaded probe‐gated SNPs based detection; hybridization of the probe and viral target on lateral flow assay (LFA) and releasing of loaded sensing materials for the direct detection of viral infection using human nasopharyngeal swab samples since LFA is easy to fabricate, relatively sensitive, suitable for clinics, and has the ability to target many analytes.^[30]^

**Figure 1:**
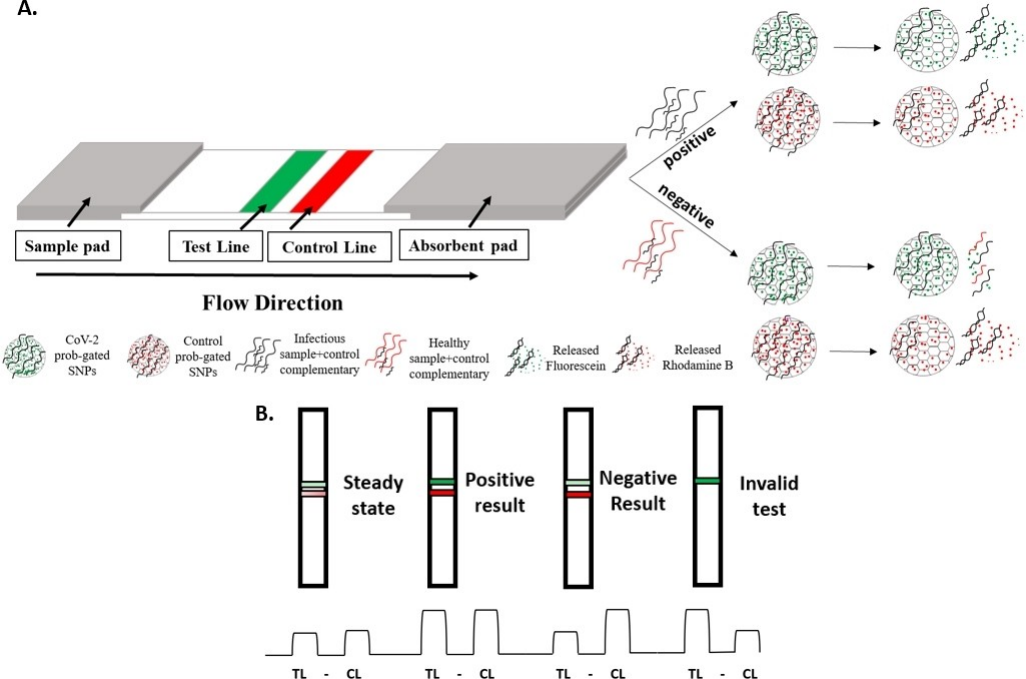
Schematic representation of the CoV‐2‐gated SNPs‐based LFA. A) Samples are added to the sample pad, then encounter the test line and the control line on the nitrocellulose membrane. Signal molecules (fluorescein and rhodamine B) will be released from the TL and CL if the sample is infected, or only rhodamine B particles will be released when it is negative (non‐infected). B) Principle of qualitative detection of viral infection. Intensities of TL (F/F_0_) and CL (R/R_0_) will increase when the sample is positive (+), only CL intensity (R/R_0_) will increase if the sample is negative (−).

In the present study, we developed a rapid, sensitive detection assay against a viral infection using dual fluorescence sensing material loaded probe‐gated SNPs. For this purpose, NSP12, NSP9, and E sites of the CoV‐2 genome were targeted for the optimization of the LFA using both synthetic oligonucleotides and human CoV‐2 virus samples obtained directly from the human nasopharyngeal swab samples. The proposed LFA mechanism here relies on hybridization between viral RNA in the samples and probes which were immobilized on the surface of the SNPs resulting in the fluorescence signal generated from the releasing of fluorescent cargo molecules. After 15 minutes of incubation, compared to the steady state, the intensity ratios of both the test line (TL) and the control line (CL) of the LFA increase if the sample is positive for the CoV‐2 virus (Figure 1A,B). Only the CL intensity increases with no change in TL if the sample is negative (Figure 1B). However, the LFA is invalid if there is no increase in CL compared to the steady state.

## Materials and Methods

### Materials and reagents

(3‐Aminopropyl) triethoxysilane (APTES), fluorescein sodium salt, Rhodamine B, and other chemicals were obtained from Sigma‐Aldrich. The LFA membranes and pads were purchased from Whatman (UK), Sample pad #07.622.30, Nitrocellulose FF120HP membrane #07.627.65, VF90 membrane #07.640.10, FF170HP membrane #07.626.70, MD100 membrane #07.643.10, FF80HP membrane #07.628.70, absorbent pads #07.623.30, and backing card #07.615.40. The specific region positive strand oligonucleotides of CoV‐2 (Viral NSP12, NSP9, and E) and complementary sequence of CoV‐2 viral sequences (Probe) were purchased from Sentromer® (Istanbul, Turkiye). The list of the oligonucleotides is given in Table 1. This study was approved by the ethics committee of the University of Health Sciences, Kartal Dr. Lutfi Kirdar City Hospital (approval number: 2021/514/202/45). The present study was performed under the Declaration of Helsinki with blind sampling.

**Table 1:** Oligonucleotide sequences of the probes and targets.

Name	Sequence (5′→3′)
NSP12_Probe_	TAC CGG CAG CAC AAG ACA TCT
NSP12_Viral_	AGA TGT CTT GTG CTG CCG GTA
E_Probe_	CGA AGC GCA GTA AGG ATG GCT AGT GT
E_Viral_	ACA CTA GCC ATC CTT ACT GCG CTT CG
NSP9_Probe_	CCT ACC TCC CTT TGT TGT GTT GTA GTA AGC TAA CGC AT
NSP9_Viral_	ATG CGT TAG CTT ACT ACA ACA CAA CAA AGG GAG GTA GG
Control_Probe_	CCT GAT AAC GAA AAC ATT AAG CCA AG
Control	CTT GGC TTA ATG TTT TCG TTA TCA GG

### Synthesis of CoV‐2‐gated SNPs

The SNPs synthesis protocol was given in our previous study.^[14]^ Briefly, the 50 mg MCM‐41 type of SNPs was stirred with 5 % one mM of acetic acid for one hour at room temperature (RT). Then, the APTES solution was added to provide aminated SNPs with a molar ratio of 0.2 and incubated on a shaker at RT overnight. The next morning, the solution was washed with 1X PBS (0.01 M phosphate‐buffered saline; NaCl‐0.138 M; KCl‐0.0027 M; pH 7.4) and centrifuged at 14,000 RPM for 5 minutes. This step was repeated three times. The 5 μg/μL amino‐modified nanoparticles were prepared in 1X PBS buffer (pH: 7.4) and loaded with 100 μM fluorescein sodium salt and rhodamine B and kept in a shaker overnight. Lastly, one μM CoV‐2 probe, shown in Table 1 (E, NSP12, or NSP9), was immobilized to the fluorescein‐loaded SNPs in the PBS buffer while control sequences were immobilized to the rhodamine B‐loaded silica nanoparticles with overnight incubation for the control line as a last step of probe‐gated SNPs. The particles were finally washed thoroughly with 1X PBS buffer three times and stored at +4 °C until their use. The fluorescein and rhodamine B entrapments were calculated from the calculation of unloaded amount Thermo Scientific™ Varioskan™ LUX‐ Fluorescence Microplate Reader (λ Ex/Em.: 460/520 nm, λ Ex/Em.: 535/600 nm respectively).

### Characterization of CoV‐2‐gated SNPs

The hydrodynamic radius and ζ potential of the SNPs were measured using Malvern Panalytical Zetasizer Nano ZS (UK). Before and after the amino‐group functionalization process (amination), NH‐ group addition on the SiO_2_ surface was characterized with Fourier transform infrared spectroscopy (Nicolet™ iS50 FTIR‐ OMNIC 0.9, ATR). The mesoporous shape of the SNPs was characterized by transmission electron microscopy (TEM) using a standard carbon grid with Jeol JEM‐2100 Plus Electron Microscopy (Japan). All SNPs were analyzed under agarose gel electrophoresis to characterize the binding affinity of CoV‐2 viral sequences and probes. Different combinations (such as SNPs, probe‐gated SNPs, and their viral complementary, and probes with viral complementary without SNPs) were loaded into the gel, and bands were analyzed using the BioRad ChemiDoc Imaging system (USA).

### LFA development and nanoparticle dispensing

The nitrocellulose reaction membrane was blocked with 1 % skim‐milk solution for one hour on the shaker at RT. After three times washing with 1X PBS, the membranes were dried at 37 °C for at least two hours and stored at +4 °C until their use. CoV‐2 probe‐gated and control‐gated SNPs were prepared using the mix of 20 % acrylamide, 0.05 M glycine, and 10 % ethanol and dispensed into the TL and CL of nitrocellulose reaction membrane using manual dispensing and dried at 37 °C for two hours, then stored at +4 °C until its use. To prepare the strips, the particle‐dispensed reaction membrane, sample pad, and absorbent pad were adhered to the backing card and cut approximately 5 mm for the measurements. The prepared strips were stored at +4 °C until their use.

### Collection of patient samples and CoV‐2 Measurement

Nasopharyngeal swab samples were collected from patients who were admitted to the Emergency Service Unit at Kartal Dr. Lutfi Kirdar City Hospital as a result of CoV‐2 symptoms or a history of contact. Nasopharyngeal swab samples were obtained using sterile synthetic fiber swabs with plastic shafts, and they were then put into vNAT® Viral Nucleic Acid Buffer (Bio‐Eksen, Turkiye) to carry out RT‐qPCR. The detection of CoV‐2 was carried out using the Bio‐Speedy® CoV‐2 Emerging Plus kit (Bio‐Eksen, Istanbul, Turkiye). This kit could identify the N D3 L mutation for the detection of the Alpha variant, the S L452R mutation for the detection of the Delta variant, and the S E484 K mutants which are primarily Gamma and Mu variants in addition to targeting the Orf1ab and N gene regions that are common in all CoV‐2 variants. To identify ORF 1 ab, the N gene of CoV‐2 and the RNase P gene, the swab samples in vNAT® buffer were vortexed for 15 seconds. Each RT‐qPCR experiment (using the Bio‐Rad CFX96 TouchTM Real‐Time PCR equipment, Bio‐Rad Laboratories) contained a non‐template negative control and a positive control. The Ct value, which is less than 32, was considered positive. The mean Ct value for positive samples was 20.7 with ranges of 14.4 to 25. Based on RT‐qPCR confirmed results, 12 positive and 12 negative CoV‐2 swab samples in vNAT® buffer were used to optimize the LFA described above. The age of patients (13 female, 11 male) in the present study ranged between 24 and 62 years old.

### LFA Validation

For the TL and CL analysis, for each probe‐gated SNP dispensed strip, 150 μL synthetic viral solutions listed in Table 1 were combined in different combinations and loaded into the sample pad. In each set of experiments, a total of four different strips were tested: 1) a combination of CoV‐2 viral solution and control solution (TL and CL specific), 2) CoV‐2 viral solution specific to TL, 3) control solution (CL specific), 4) PBS buffer solution as a negative control. The TL and CL of strips were measured without any sample to evaluate nanoparticle long‐term stability (2^nd^‐ 8^th^‐ 15^th^‐ 30^th^‐day measurement). The TL and CL of strips were also measured with the sample after the measurement of one week to observe signal generation location. To adjust assay measurement time, after sample addition, strips were measured at 5, 15, and 30 mins. Moreover, the best pH for the system has been analyzed from 6 to 9 pH using synthetic viral complementary. The LOD was also measured using synthetic viral complementary ranging from 500 nM to 100 fM. For the assay performance evaluation, a dilution range of 1 : 10^1^ to 1 : 10^4^ was used for human samples in order to find the optimal concentration. Rest of the experiments, human samples with a dilution rate of 1 : 10^3^ were loaded to the assay strip with a fixed incubation time of 15 minutes (n=12). The 1 μM control sequences were added to each human sample for the CL as a running buffer. The Zeiss Discovery V.20 microscope red/green filters were used for imaging while Image J was used to quantify each LFA strip. The sensitivity, specificity, and accuracy were calculated using the following formulas [Eq‐ (1–3].

(1) $$\mathrm{Sensitivity}\;\left(\%\right)=100^{*}\left(\mathrm{True}\;\mathrm{positive}/(\mathrm{True}\;\mathrm{positive}+\mathrm{False}\;\mathrm{negative})\right)$$

(2) $$\mathrm{Specificity}\;\left(\%\right)=100^{*}\left(\mathrm{True}\;\mathrm{negative}/(\mathrm{True}\;\mathrm{negative}+\mathrm{False}\;\mathrm{positive})\right)$$

(3) $$\mathrm{Accuracy}\;\left(\%\right)=100^{*}\left(\mathrm{True}\;\mathrm{positive}+\mathrm{True}\;\mathrm{negative})/(\mathrm{Total}\;\mathrm{number}\;\mathrm{of}\;\mathrm{samples}\right)$$

### Statistical analysis

The data was displayed as mean±standard deviation and at least three independent measurements were performed for each experiment. An unpaired and paired Student's t‐test was used to evaluate assay performance during the optimizations with a confidence interval of 95 %.

## Results and Discussion

Detection of rapid viral infections, especially positive‐stranded RNA virus infections, is crucial for controlling the spread of viral infection throughout the world since viruses can rapidly mutate into different variations which make fighting against this viral infection challenging. The recent CoV‐2 pandemic has shown us the importance of the diagnostic rapid screening process which is essential to control infectious diseases because efficient treatment against viral diseases is usually unavailable. However, if there is a universal‐modifiable diagnostic assay available to detect the new variants of the virus, this may give us more time to better understand the disease and use proper medications or treatments. Hence, there is still an urgent need for a modifiable rapid assay for CoV‐2 detections and other positive stranded viral infections, including, *Picornaviridae*, *Caliciviridae*, and *Hepeviridae* families.^[31]^ To address this problem, we designed a fluorescence mode lateral flow assay using fluorescein and rhodamine B‐loaded probe‐gated silica nanoparticles. The mechanism relies on fluorescein releasing in the presence of hybridizing viral genome, while rhodamine B releasing will be obtained from the control probe for the assay validation.

### Synthesis of SNPs

Single‐stranded oligonucleotides are commonly preferred targeting and gated material since it is easy to produce, inexpensive, and sensitive.^[32]^ The single‐stranded oligonucleotides encapsulate the specific cargo molecules inside the pores of nanoparticles using its active phosphate group of backbone using aminated SNPs.^[33]^ Therefore, after the synthesis and functionalization of mesoporous SNPs, fluorescein and rhodamine B as a sensing material were loaded into mesoporous silica nanoparticles and encapsulated by single‐stranded probes via electrostatic interaction between amino groups on the particle surface and the phosphate group of oligonucleotide‐probes.

### Characterization of the SNPs

**Figure 2:**
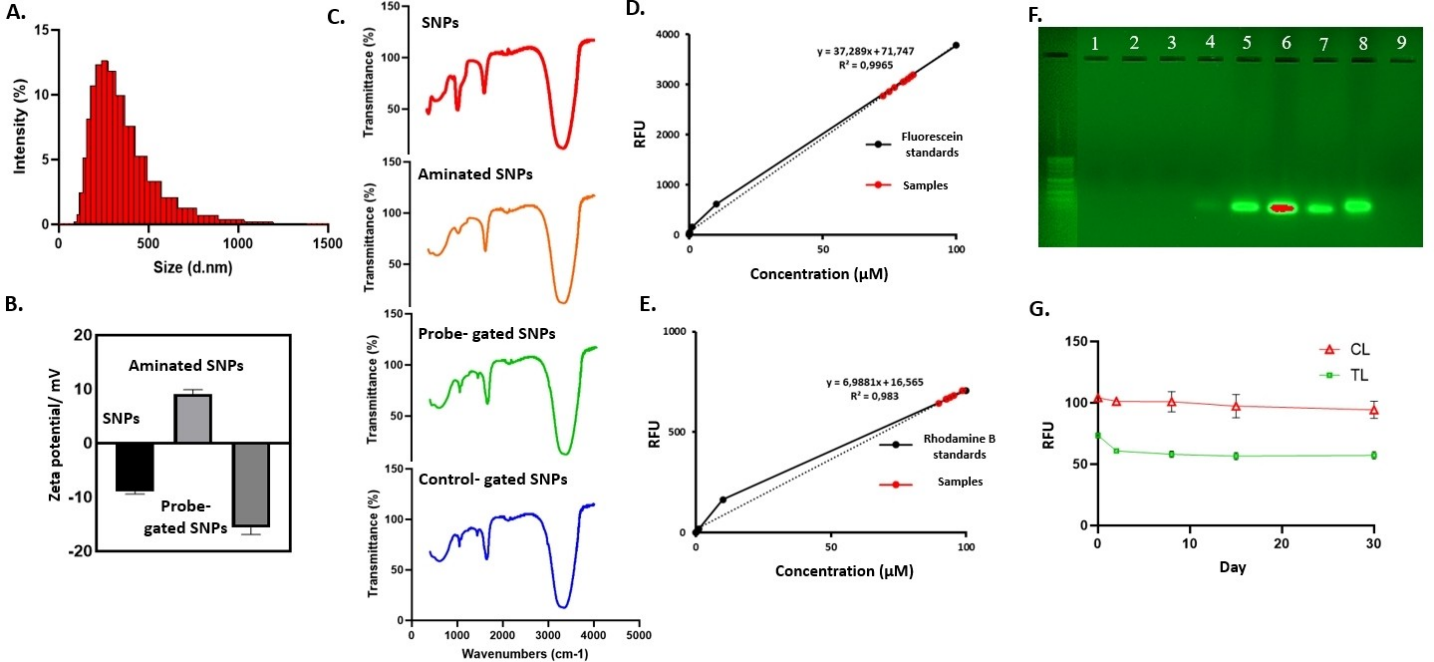
Characterization of SNPs. A) Size of the viral probe‐gated SNPs was measured using DLS and it was 284.8±21.0 nm. B) The zeta potential of the nanoparticles was −8.86±0.54 mV and +9.12±0.78 mV before‐after amination, and probe‐gated SNPs were −15.7±1.27 mV. C) Viral probe‐gated SNP bands were detected using FTIR‐ATR. D and E) Fluorescein and Rhodamine B entrapment inside the mesoporous of SNPs. F) Viral and its probe sequences (Table 1) were measured in agarose gel electrophoresis. (Lane 1: SNPs, 2: NSP12‐gated SNPs, 3: NSP12‐gated SNPs and its viral complementary, 4: NSP12 probe and SNPs, 5: Control probe, SNPs and its complementary, 6: E gene probe and its viral complementary, 7: NSP12 probe and its viral complementary, 8: NSP9 probe and its viral complementary, 9: NSP12‐gated SNPs and PBS as a negative control.) G) Time‐course monitoring of fluorescence signal at the test line and control line. (RFU: real fluorescence unit).

The sizes of SNPs were measured as 189.5±30.5 nm (Figure 2A). Although this size of SNPs is high, it is suitable for LFA development. The SNPs used for LFA development in the literature are approximately the same size, or even higher.[^34^, ^35^] The ζ‐potential was −8.86±0.54 mV before the amination process, while it was +9.12±0.78 mV after the amination process. The ζ‐potential of viral probe‐gated SNPs was changed to −15.7±1.27 mV (Figure 2B). The amination process was also evident by FTIR analysis (Figure 2C). The peaks around 3600 cm^−1^ and 1052 cm^−1^ after amine functionalization, were assigned to N−H and C−N bending vibrations via primary amine formation. In addition, around 1439 cm^−1^ and 1597 cm^−1^ peaks of probe‐gated SNPs confirmed that there is a strong N−O stretching, which originates from probe oligonucleotides on the surface of the mesoporous SNPs. Loaded fluorescein and rhodamine B concentrations were measured by creating a standard curve (Figure 2D,E). The amount of fluorescein loaded in the probe‐gated SNPs was calculated as 4.19 μmol ⋅ g^−1^, while rhodamine B 1.23 μmol ⋅ g^−1^into the mesoporous of SNPs. This loading amount proves that SNPs have an excellent loading capacity.^[36]^

The bare SNPs, probe‐gated SNPs were tested for producing specific fluorescent signal in the presence complementary genomic sequences of the virus. The electrophoretic analysis was used to assess the detachment of probes from silica surface upon hybridization with its complementary sequences. The supernatants of hybridization experiments shown in Figure 2F. There was no band detected for the SNPs, NSP12‐gated SNPs, NSP12‐gated SNPs and its viral complementary, also NSP12‐gated SNPs and PBS group (lane 1‐2‐3‐9). In addition, there was a band to confirm double‐strand formation for oligonucleotide‐probe analysis; control probe, SNPs and its complementary, also three CoV‐2 probes and their viral complementary (lane 5‐6‐7‐8). Interestingly, there was also a slight band for NSP12 probe and SNPs (lane 4) which confirms unconjugated single‐stranded DNA, while there was no band for conjugated DNA (Figure 2F).

The probe‐gated SNPs were then dispensed into nitrocellulose membranes on TL and CL. Then, the strips were measured without any sample application to analyze the stability of fluorescence ability sensing material up to 30 days. The strips were stable with no change in the fluorescence properties of the particles even after 30 days of the preparation (Figure 2G). There was a slight decrease in fluorescence signal on day 2 (Figure 2G). This could be due to the particles being slightly absorbed by exposure to the laser during previous measurement.

### LFA Optimization

Five types of nitrocellulose reaction membranes with different flow rates were analyzed and the FF120HP membrane among all generated a better signal for SNP design (data not shown). The NSP9‐gated and E gene‐gated strips did not give significant results for target detection. This could be due to the size of the sequences since the long bp gate may not recognize its target sequences. Also, the long‐size sequence did not flow along the membrane in the viral synthetic solution within 15 min (Figure S.1A in Supporting Information). A study showed that fluorometric LFA using 64 bp aptamer detects creatine kinase MB in 20 min.^[37]^ In terms of binding affinity, long bp oligonucleotides or aptamers require a long incubation time. In our study, NSP12‐gated SNPs‐based LFA had high binding affinity compared to NSP9 and E gene targets within 15 min. In this context, a study by Chang et al. reported the successful application of DNA sensors using a 25‐base pair DNA‐gated fluorescent sensor based on PEI, chitosan, and silicon dioxide.^[38]^ Their result supports our findings since we also used sequences in the size of 25 bp for NSP12‐gated SNPs‐based LFA. In addition, we also optimized the detection time for the viral infection using NSP12‐gated SNPs‐based dispensed LFA strips. The target detection time was determined as 15 minutes since there was not sufficient signal after 5 minutes and the generated signal at 30 minutes was similar to that of at 15 minutes (Figure S.1B). Therefore, 15 minutes was decided to be the optimum time point for the detection. Our findings are similar to the ones in the literature (Table 3). For example, in other studies, the quantum dots and rhodamine detection time points in rapid assays are also reported as 15 minutes.[^39^, ^40^] On the other hand, Ozalp et al. reported assay time as 3–5 minutes using one single sensing material, rhodamine, which significantly reduces the detection time points.^[28]^ Therefore, 15 minutes seems to be the optimum time point for detection using a fluorescence nanoparticle system.

**Figure 3:**
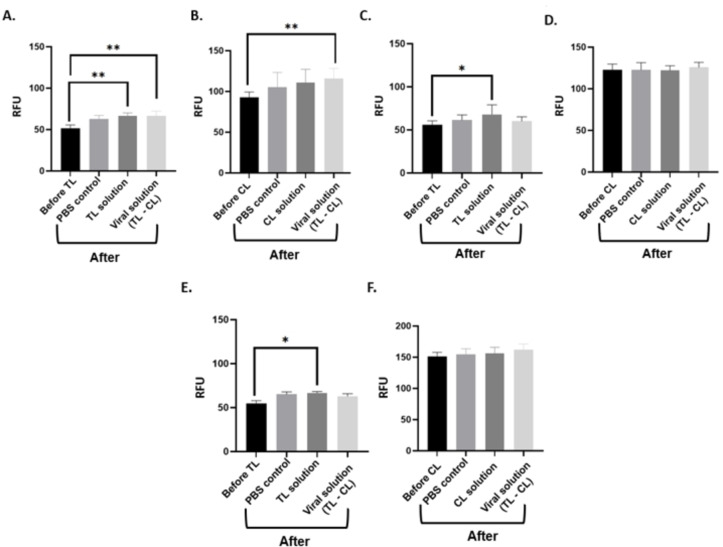
Synthetic sample evaluation of the strips. A) TL of NSP12‐gated SNPs based LFA B) CL of NSP12‐gated SNPs based LFA C) TL of E gene‐gated SNPs based LFA D) CL of E gene‐gated SNPs based LFA E) TL of NSP9‐gated SNPs based LFA F) CL of NSP12‐gated SNPs based LFA responses against different synthetic sample solutions after 15 minutes of application. (RFU: real fluorecence unit, n=3, *p<0.05, **p<0.001).

To evaluate assay performance, the NSP12, NSP9, and E gene‐gated SNPs‐based LFA strips were measured using four different synthetic sample combinations (Figure 3). The TL‐generated signal, which was around 24.5 %, was higher than the steady‐state TL signal for the CoV‐2 viral solution specific to TL (Figure 3A, p<0.001). In addition, the TL‐generated signal was similar to that of CoV‐2 viral and CL (TL and CL specific) solution which confirmed the specific recognition in TL when NSP12‐gated strips were considered (Figure 3A). For the CL, the signal generation was increased by 7.1 % when CL solution measurement was compared to before CL (Figure 3B, p<0.001). The combined application of viral solution (TL‐CL) increased the binding affinity of control probe‐gated SNPs in CL. On the other hand, the E gene and NSP9‐gated SNPs generated less signal compared to that of NSP12‐gated SNPs (Figure 3C,D,E,F, and Figure S.2, p<0.05). Especially, NSP9 and E gene‐gated SNPs generated poor signals for the CL (Figure S.2). There was no statistically significant difference between the groups for the E gene and NSP9‐gated SNPs (p>0.05). After all these analysis results, NSP12‐gated SNPs were selected for further analysis in LFA to increase assay performance.

To indicate the target analyte detectability of the NSP12‐gated SNPs‐based LFA, the LOD was calculated as 0.73 pg ⋅ mL^−1^ (Figure S.3). According to fluorescence‐based detection methods, the LOD using NSP12‐gated SNPs‐based LFA is sufficient for the detection of viral infection compared to the literature. For instance, Ozalp et al., J. Zhang et al., and Molulahoum et al. reported the LOD ratios as a 69 μM, 0.63 ng ⋅ mL^−1^, and 0.53–0.31 ng ⋅ mL^−1^ using fluorescence‐based detection methods.[^28^, ^37^, ^40^] The LOD value of dual‐mode fluorescence detection assay is not as sensitive as compared to our assay, such as Climent et al. showed that the LOD value of different fluorescence sensing material loaded antibody gated silica nanoparticle is around μg ⋅ L^−1^ rates in rapid period.^[41]^ Considering the difficulty of creating a design with fluorescent particles, this result is promising in a short time like 4 minutes, but in this study, we obtained a more sensitive LOD value by using two different fluorescent sensing molecules. On the other hand, C. Wang, Cheng, et al. reported the LOD ratio as 1 pg ⋅ mL^−1^ in 30 min, using fluorescence‐based detection based on magnetic quantum dots which makes the system more specific and sensitive.^[42]^ Comparing the detection time points, probe‐gated SNPs detect its viral target analyte in 15 min with a 0.73 pg ⋅ mL^−1^ LOD value which shows this assay applicability. As seen in our previous study, the LOD ratio using silica nanoparticles‐based detection of viral infection could be 48 fM in the tube in vitro conditions.^[14]^ However, in the LFA system, this situation is more difficult, and the LOD value we have shown in this study is sufficient for the detection of viral infection, according to the detection of viral infection sensitivities. Moreover, this study is a proof‐of‐concept for the detection of viral infections. It is very difficult to adapt this concept in clinics. Therefore, in the current study, we showed not only assay sensitivity but also practicality.

**Figure 4:**
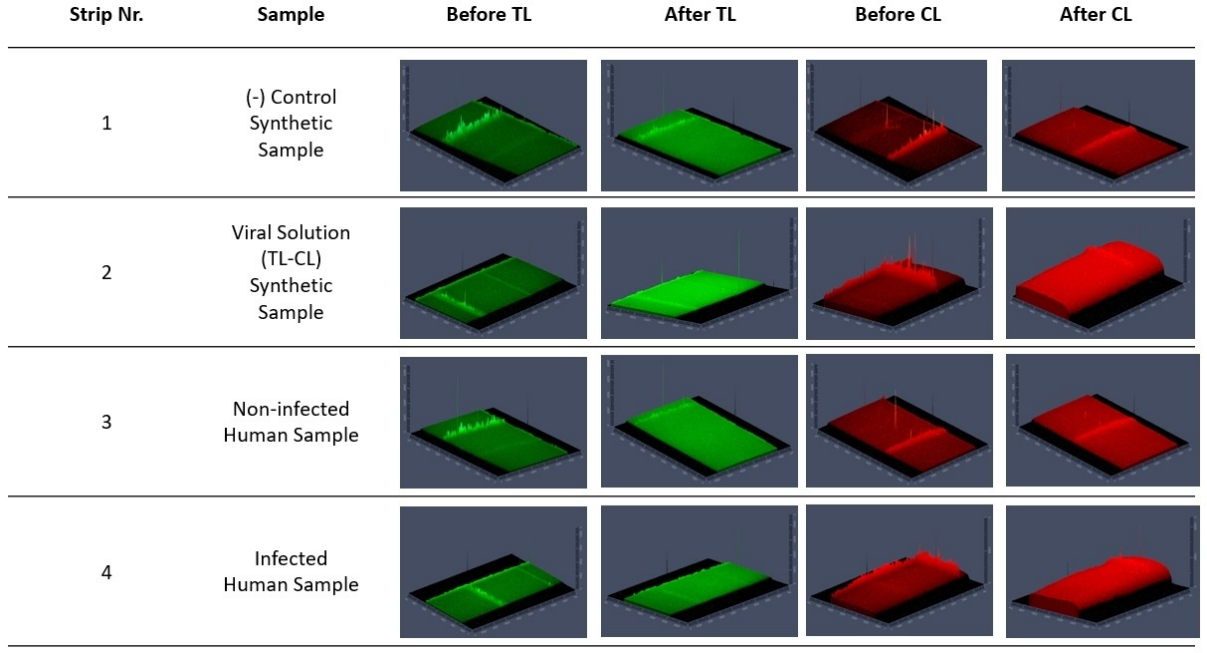
Fluorescence scans of NSP12‐gated SNPs‐based LFA under green and red fluorescence filters.

To visualize and evaluate NSP12 gated SNPs‐based LFA, each strip was analyzed under green and red fluorescence filters during the assay evaluations (Figure 4, Figure S.4). The TL generated less signal compared to CL for each measurement. TL and CL signal intensities of the first and third strips were changed after the application of neither negative control nor non‐infected human swab samples. However, the TL and CL signal intensity of the second and fourth strips were changed after the application of either the viral solution or the infected human swab sample. Indeed, the CL created a higher signal which looked like an airbed than the steady state (Figure 4, strips no 2 and 4, after CL). The LFA strip images indicated that the assay results could be interpreted easily, even so, the strip intensities were quantified and formulated. The F/F_0_ and R/R_0_ ratios were used for the calculation and evaluation of TL and CL, respectively. The F/F_0_ ratio explains the result with cut‐off values as positive if the value is bigger than 1.2 (>1.2) or negative if the value is smaller than 1.2 (<1.2) while the R/R_0_ ratio should be between 0.9 and 1.1 for the validation of the assay strip. Furthermore, the released fluorescein and rhodamine B from used strips were accumulated in the absorbent pad one week after analysis (Figure S.5).

### Clinical sample measurement by probe gated LFA

**Figure 5:**
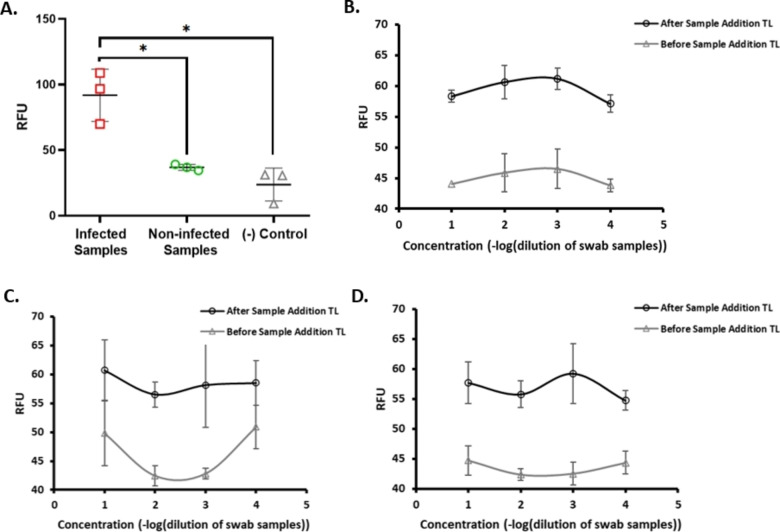
The measurements at pH: 7.4 (A) and measurements of the limit of detection (B,C,D). A) Analysis of infected, non‐infected, and negative control samples at pH 7. B) NSP12‐gated SNPs‐based LFA, C) NSP9‐gated SNPs‐based LFA, D) E gene‐gated SNPs‐based LFA; TL analysis to find optimum detection limit of human swab samples (n=3, *p<0.05), (Circular black line: after sample addition TL, triangle grey line: before sample addition TL for Figure B, C, D).

The optimum pH of the strip analysis revealed that pH 7.4 is the optimum pH condition for the clear separation of infected, non‐infected, or negative control from each other compared to the pH 6, pH 8, and pH 9 (Figure S.5, p<0.05). The CoV‐2 viral infected samples had a higher signal at pH 7.4, pH 8, and pH 9 compared to that at pH 6, while non‐infected and negative control samples had a similar amount of signal at pH 7.4. But there was a high fluorescence signal for non‐infected samples at pH 8 and pH 9 while there was less signal for infected samples. Moreover, the signal was similar at pH 6 and pH 7.4 for non‐infected samples, but it was not similar for the infected samples at pH 6 and pH 7.4. Therefore, pH 7.4 was chosen as the optimum pH for the detection of viral infection (Figure 5A). In this context, similar to the current study, Qui et al. and Chang et al. also reported pH 7.4 as an optimal pH for bio‐responsive fluorescence detection using quantum dots and silica nanoparticles.[^38^, ^43^]

To evaluate the minimum detection limit for human swab samples without any processing protocols, the samples were diluted within a range from 1 : 10^1^ to 1 : 10^4^ for NSP12, NSP9, and E gene‐gated SNPs‐based LFA (Figure 5B,C,D). The NSP12‐gated SNPs‐based LFA generated a higher signal at TL using the concentration of 1 : 10^3^. This could be due to the concentration of 1 : 10^1^ may have a high concentration of lysis buffer to release viral load and detergents which vNAT® Viral Nucleic Acid Buffer (Bio‐Eksen, Turkiye) has. As a result of it, a high concentration of these ingredients could degrade the structure of the nanoparticle system. Direct dilution of vNAT solution is one of the advantages of viral infection detection. This process also makes the assay sensitive for detection. On the other hand, one study reported that silica‐core@dual quantum dot shell nanotags detected its antibody target with a LOD 1 : 10^7^ which is based on one single fluorescence material.^[39]^ Although their dilution rate is higher than what we used in the current study, excitation and emission separation between TL and CL during measurements using two different fluorescence sensing materials in mesoporous silica nanoparticles were perfectly obtained.

**Figure 6:**
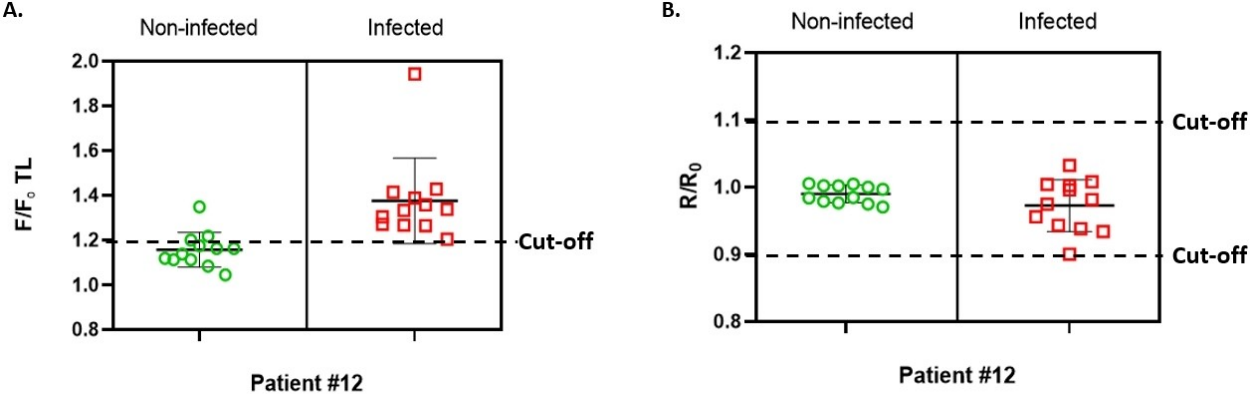
Evaluation of efficacy of the strips using NSP12‐gated nanoparticles. A) F/F_0_ signal analysis B) R/R_0_ signal analysis with specific cut‐off values (positivity cut‐off is 1.2; validation cut‐off is between 0.9 and 1.1, n=12).

We also evaluated the accuracy of the probe‐gated SNPs‐based LFA design using infected or non‐infected clinical human samples which were already confirmed with RT‐qPCR (n=12). The samples were diluted to a concentration of 1 : 10^3^ and tested using NSP12, NSP9, and E gene gated LFA strips (Figure S.7). The TL F/F_0_ signals were increased for all probe‐gated SNPs since CL R/R_0_ signals were only increased in NSP12‐gated strips, which were a sufficient signal for the validation of the assay results. According to F/F_0_ and R/R_0_ cut‐off values, the NSP12‐gated SNPs‐based LFA strips were in the range for infected and non‐infected samples (Figure 6A, B and Table S1). All strips were valid; infected samples were tested correctly while three non‐infected samples were a false positive signal. E gene‐gated strips generated high signals according to the F/F_0_ ratio for infected or non‐infected samples, however, all strips were not valid according to R/R_0_ ratio (Figure S.8A, B, Table S2). In contrast, the NSP9‐gated strips were valid except for one, however, it generated false positive (n=3) and false negative (n=6) signals according to the F/F_0_ ratio (Figure S.8 C, D, Table S3). The results indicated the successful quantification of NSP12‐gated SNPs‐based LFA strips and optimum design with a sensitivity of 100 %, specificity of 83.3 %, and accuracy of 91.7 % for the detection of viral infection in clinical samples (Table 2). On the other hand, E gene‐gated SNPs‐based design had an accuracy of 87.5 % which is lower than that of NSP12. These ratios demonstrated that fluorescence probe‐gated SNPs‐based LFA is sensitive and substantially outperforms standard rapid diagnostic assays which are used in the market currently. According to our previous study, we increased the sensitivity from 84 % to 100 % using two fluorescence‐based sensing materials in LFA. This sensitivity is also higher than RT‐qPCR as a gold standard for CoV‐2 infection detection. The sensitivity and specificity values obtained are sufficient for the detection of viral infections such as CoV‐2 and could be easily modified for new viral infections in the future. Considering these results, it has been shown that probe‐gated SNPs based LFA are as sensitive as RT‐qPCR or even more so and could be used clinically.

**Table 2:** Analytical properties of the probe‐gated SNPs‐based LFA.

	Clinical Positive Sample	Clinical Negative Sample	Sensitivity	Specificity	Accuracy
Sample Quantity	12	12			
NSP12‐gated SNPs	12	10	100	83.3	91.7
NSP9‐gated SNPs	6	9	50	75	62.5
E gene‐gated SNPs	12	9	100	75	87.5

In the current study, we aimed to develop fluorescence sensing material probe‐gated SNPs based LFA for the detection of viral infections. In this context, the number of fluorescence‐based nanoparticle LFA designs has increased over the five years. Recent studies have reported that fluorescence‐based LFA designs (Table 3) using silica nanoparticles or other nanoparticle designs have high sensitivity for target detection since the LFA is a good candidate for rapid point‐of‐care diagnostic assays as it is easy to fabricate, user‐friendly, and low‐cost. In these assays, the rhodamine, fluorescein, FITC, and other sensing materials have commonly been chosen as signal generator molecules and loaded into carrier molecules like mesoporous silica nanoparticles or quantum dots and then gated by single‐stranded DNA probes, aptamer, or antibodies in a different field.[^28^, ^37^, ^39^, ^40^, ^41^, ^42^, ^44^, ^45^]

**Table 3:** Comparison of the results from the literature for various fluorescence‐based approaches.

Method	Materials	LOD	Assay Time	Ref.
Fluorescence	Rhodamine loaded silica particles	69 μM	3–5 min	[28]
Fluorescence	Silica‐core@dual quantum dot Shell nanotags	IgM/IgG (1 : 10^7^ dilution)	15 min	[39]
Fluorescence	FNP and Fluorescent‐labelled antibody	1,000 TU mL^−1^	15 min	[44]
Fluorescence	Aptamer‐based	0.63 ng ⋅ mL^−1^	20 min	[37]
Fluorescence	Rhodamine B‐loaded polymersomes	0.53 and 0.31 ng ⋅ mL^−1^	5–10 min	[40]
Fluorescence	Magnetic quantum dot	1 pg ⋅ mL^−1^	30 min	[42]
Fluorescence Dual mode	2,7‐dichlorofluorescein and sulforhodamine B loaded antibody‐gated silica nanoparticles	2.9±0.5, 0.9±0.2 and 9.2±4.3 μg ⋅ L^−1^	>4 min	[41]
Fluorescence and absorbance Dual mode	G‐quadruplex/N‐methylmesoporphyrin IX (G4/NMM) and 3,3′,5,5′‐tetramethylbenzidine (TMB)	0.195–25 ng ⋅ mL^−1^, 0.049–1.563 ng ⋅ mL^−1^	5 min	[45]
Fluorescence mode	**Fluorescein and Rhodamine B loaded probe‐gated silica nanoparticles**	**Synthetic probes (0.73 pg ⋅ mL^−1^)** **Human Swab Samples** **(1 : 10^3^** **dilution)**	**15 min**	**The Current Study**

Considering the chemical reactions in the field of nanomaterials, as each system has different properties within itself, studies are carried out to make life easier, especially in clinical use. The small molecule targeting used in this study has many advantages and high sensitivity, especially in the field of health. For instance, in one study, an LFA for HIV‐1 RNA was developed using oligonucleotides conjugated gold nanoparticles.^[46]^ Small molecule targeting is promising in difficult‐to‐detect target analytes such as HIV‐1 virus. Since the CoV‐2 virus also mutates rapidly, its detection has been difficult. Therefore, targeting a modifiable small molecule is an advantage. Furthermore, although small molecule targeting is a sensitive method, most studies offer pre‐processing before direct application to the LFA. Before the samples are applied, the target analyte is amplified by specific amplification. In this study, we demonstrated small molecules targeting silica nanoparticles in high LOD and sensitivity without the need for such amplification for our design.

Fluorescence signal molecules are the strongest reporter molecule in the LFA system with a sharp distinction between excitation and emission wavelength. Fluorescein could be used in the LFA system as a labeling molecule like FITC and a direct detection molecule like releasing a reporter.[^28^, ^41^, ^47^, ^48^] These systems rely on FITC signaling with a target analyte reaction in TL or CL. Moreover, the rhodamine B molecule gives a detectable signal in the LFA system, especially for polymersomes with a high encapsulation efficiency.^[40]^ Moulahoum et al. showed that not only rhodamine B but also fluorescein molecule has small size and high encapsulation efficiency as loading reporter molecule in LFA with a response of 94 % for the sandwich assay and 97 % for the competitive LFA compared to other reporters such as xylenol orange, ponceau S, crystal violet, etc.^[40]^ Therefore, fluorescein and rhodamine B reporter signal molecules are good candidates in LFA.

In the literature, similar to our current study design, there is one recently published study, in which fluorescein and rhodamine molecules were also used.^[41]^ In the study, they reported that releasing these two sensing materials from the beginning of the nitrocellulose membrane to the inside of bare mesoporous silica nanoparticles which were dispensed in TL and CL. They also reported that released sensing materials were accumulated into the bare mesoporous silica nanoparticles for the detection of PETN. But the challenging part of their techniques is the accumulation of released sensing molecules from other parts of the LFA like the conjugation pad, or nitrocellulose membrane is difficult to produce. Thus, it is not applicable to use in the market and mass production. In addition, in the study conducted by Climent et al., the dispensed molecules could be mixed in the nitrocellulose membrane or dispersed. Moreover, the dispensed molecules may not recognize their targets due to their short size. Therefore, we propose a fluorescence LFA design by dispensing fluorescein‐loaded SNPs in TL, and rhodamine‐loaded SNPs in CL for the rapid detection assay, which is more applicable according to the literature. We reveal that the sensitivity and modifiability of the method are not only for the detection of CoV‐2 infection but also for the detection of other viral infections. In this context, there are studies similar to the current study which reported a practical sample detection method using fluorescence and absorbance dual mode to detection of ochratoxin A which indicates material science importance in nanomedicine.^[45]^ Also, in another study, CoV‐2 detection was reported using direct and enrichment dual‐mode fluorescence lateral flow immunoassay magnetic quantum dots.^[42]^ These studies also revealed the applicability of dual fluorescence detection systems in a variety of applications including health clinics since they are sensitive, rapid, and more economical. In summary, our fluorescence mode detection probe‐gated SNPs‐based LFA nanoparticle design is sensitive, rapid, and modifiable compared to the rapid assays which are used in the market currently.

## Conclusions

In the current study, we developed a highly sensitive and rapid point‐of‐care diagnostic assay for the detection of viral infections like the recent CoV‐2 pandemic. Using this signal generation method by fluorescein and rhodamine B, the CoV‐2 infection could be detected directly from nasopharyngeal swab samples within 15 minutes with a sensitivity of 100 %, specificity of 83.3 %, and accuracy of 91.7 % compared to RT‐qPCR since the gold standard has less sensitivity (~82 %) and lots of limitations such as, time, cost and difficult procedure. The fluorescence nanoparticle system is highly sensitive and quantitative detection could be obtained within a short period of time. For the detection of CoV‐2 viral infection and other positive‐stranded RNA viruses, the developed point‐of‐care assay could be preferred due to its rapid, easy‐to‐use, and universal‐modifiable, and it is suitable for new viral pandemics. Therefore, the current detection method for viral infections could be an alternative one to those used in the market currently.

## Supporting Information Summary

The Supporting Information includes strip measurements according to the time and re‐measurements of the strip, one week. The released sensing material accumulated at the absorbent pad after one week. We showed that 15 minutes is optimum for the detection of viral infection using the proposed design. In addition, a comparison of different gene targets of CoV‐2 detections using TL and CL was given in the Supporting Information. NSP12‐gated SNPs‐based LFA strips generated higher signals than other groups, which we selected as an optimum design for viral infections. The calculation way of F/F0 and R/R0 were given with a fluorescence scan of strip images. The optimum pH of the lateral flow assay was determined as pH:7, and other pH responses were given in the summary. After the optimization, different CoV‐2 gene responses using human swab samples were calculated, and we showed that NSP‐12 gated SNPs have higher intensity values to separate the infected and non‐infected groups. Lastly, the raw data of F/F0 and R/R0 measurements were given for each gene target of CoV‐2.

## Conflict of interest

The authors declare that they have no known competing financial interests or personal relationships that could have appeared to influence the work reported in this paper.

1

## Supporting information

As a service to our authors and readers, this journal provides supporting information supplied by the authors. Such materials are peer reviewed and may be re‐organized for online delivery, but are not copy‐edited or typeset. Technical support issues arising from supporting information (other than missing files) should be addressed to the authors.

Supporting Information

## Data Availability

The data that support the findings of this study are available from the corresponding author upon reasonable request.
