# Supplementary material for: Direct Detection of Viral Infections from Swab Samples by Probe‐Gated Silica Nanoparticle‐Based Lateral Flow Assay
Source: ChemistryOpen. 2023 Oct 12;13(2):e202300120. doi: 10.1002/open.202300120 (PMC10853071; doi:10.1002/open.202300120)
Supplement: Supplementary file 1 — Supporting Information [file OPEN-13-e202300120-s001.pdf]

ChemistryOpen

Supporting Information

Direct Detection of Viral Infections from Swab Samples by Probe-Gated Silica Nanoparticle-Based Lateral Flow Assay

Dilara Buse Durdabak, Soner Dogan, Serap Demir Tekol, Caner Celik, Veli Cengiz Ozalp, and Bilge Guvenc Tuna*

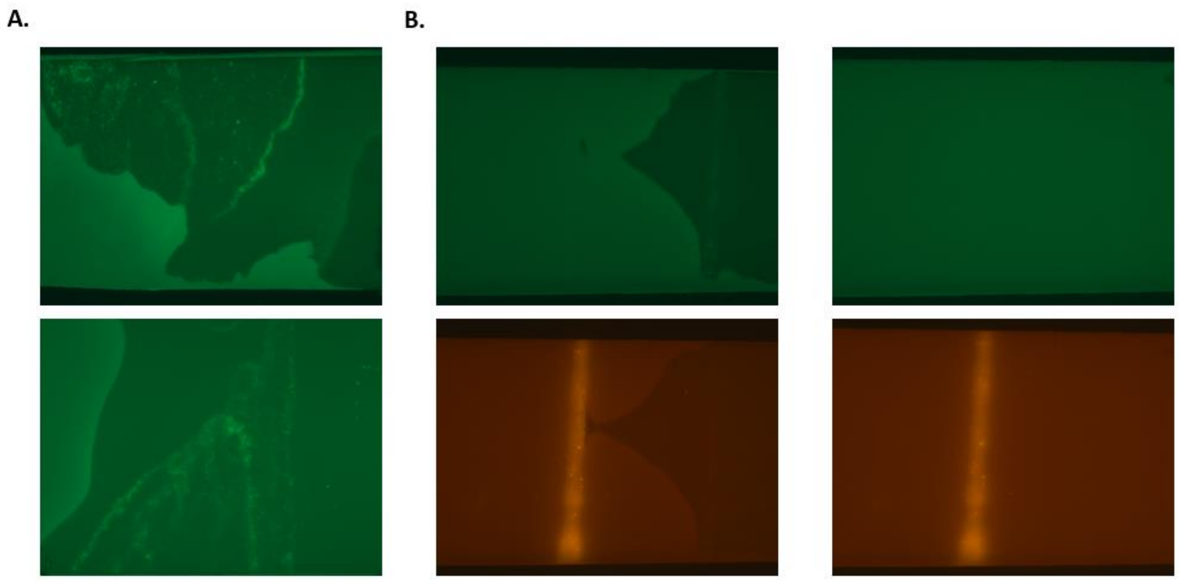

Figure S.1. Strip images **A)** E and NSP9-gated SNPs-based LFA flow inhibition. **B)** The flow is finished for NSP12-gated SNPs-based LFA in 15 min. Left side (5 min), right side (15 min).

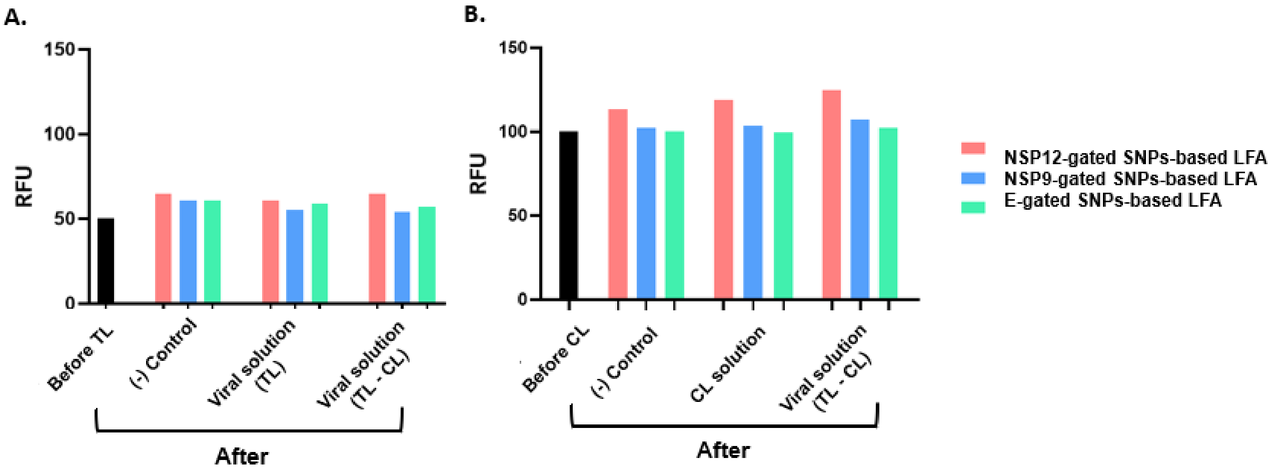

Figure S.2. Different probes-gated SNPs–based LFA responses against several combinations of synthetic sample solution; PBS as a negative control, Viral solution for TL, Viral solution for TL-CL, and CL solution. **A)** TL, **B)** CL analysis. NSP12-gated SNPs-based LFA strips generated higher signals than other groups, which we selected as an optimum design for viral infections.

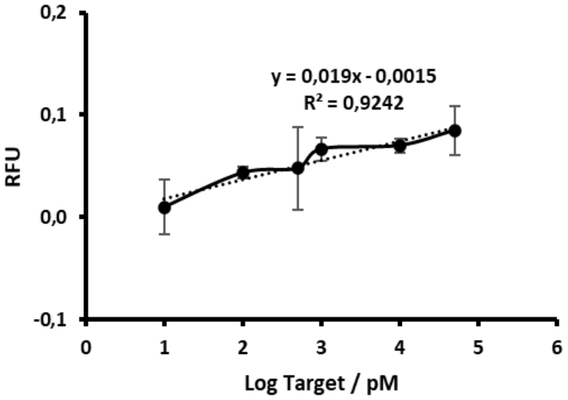

Figure S.3. LOD Analysis of NSP12-gated SNPs- based LFA. It was calculated as 0.73 pg·mL^{-1} using the 3σ method.

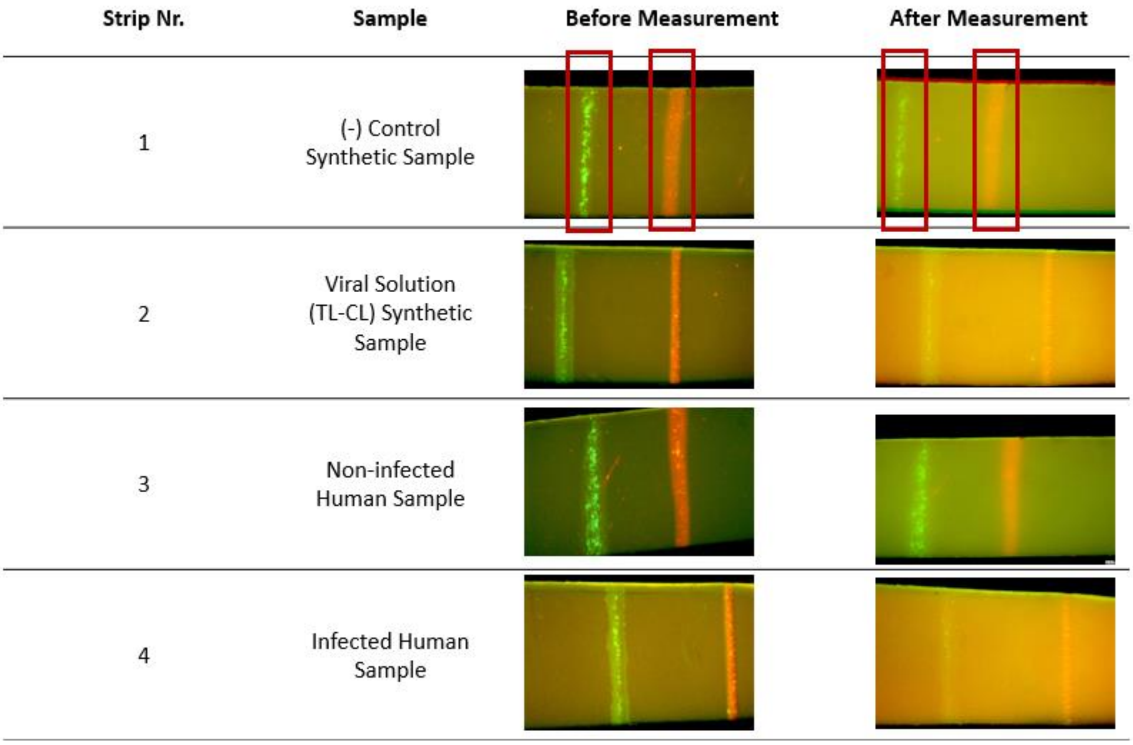

Figure S.4. The probe-gated SNPs-based LFA strip responses for negative control, viral solution for TL-CL, non-infected and infected human swab samples. For negative samples, it is seen that TL and CL intensities are almost the same when viewed with the naked eye (rows 1-3). The situation is different for positive samples. Looking at TL and CL (rows 2-4), it is seen that the particle intensities have decreased.

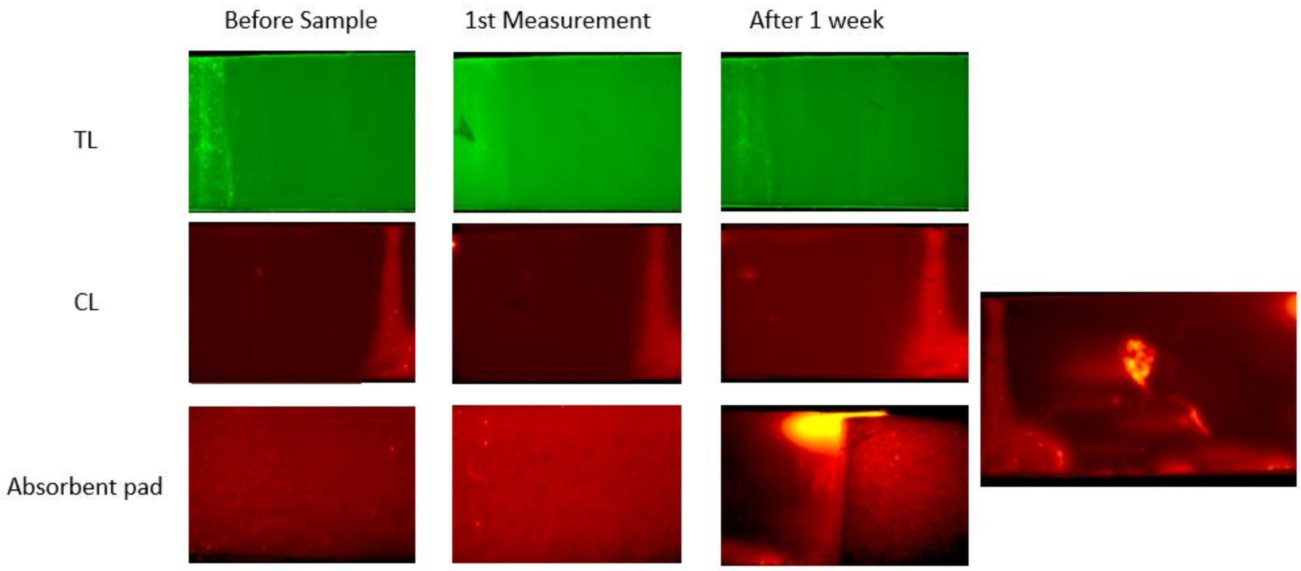

Figure S.5. Test stability of viral solution (TL+CL) after measurement. The released sensing material accumulated at the absorbent pad after one week.

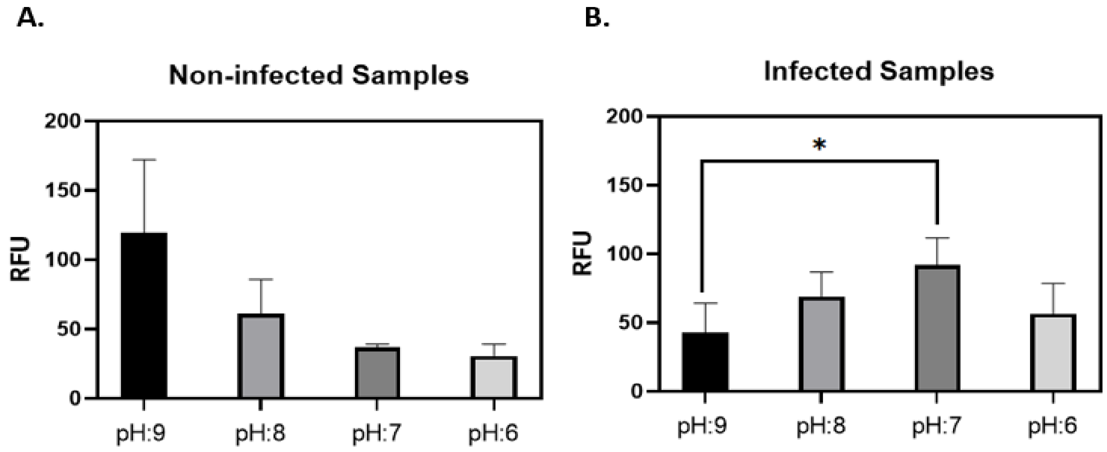

Figure S.6. pH optimization. **A)** Non-infected human swab samples, **B)** Infected human swab samples pH optimization between pH 9-6 since pH 7 gave an optimum fluorescence intensity in both TL and CL measurements.

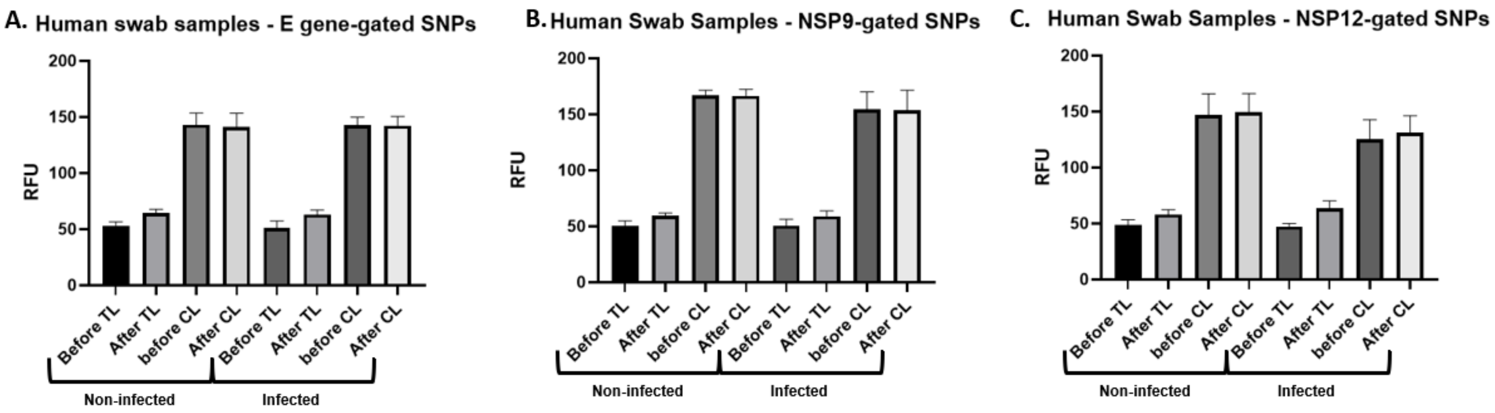

Figure S.7. Human swab samples TL and CL signal responses for **A)** E-gated SNPs **B)** NSP9-gated SNPs **C)** NSP12-gated SNPs based LFA. The before-after measurements of CL were almost the same for the three groups since the TL intensity difference was higher before-after measurements for positive samples. The intensity difference in the E gene-gated and NSP9-gated SNPs-based LFA was not adequate for test meanings. In contrast, NSP12-gated SNPs-based LFA strips have higher intensity to separate the infected and non-infected groups.

Calculation of F/F_0 and R/R_0 during measurement

The probe-gated CoV-2 strips were measured before and after the analysis of the real samples. F_0 refers to before TL intensity, and F refers to after TL measurement. R_0 refers to before CL intensity, and R refers to after CL measurement.

The formula is that:

- $Test\ Result\ (TL) = \frac{F}{F0}$; If this ratio> 1.2, the result is positive, if not it is negative.
- $Test\ Result\ (CL) = \frac{R}{R0}$; If this ratio is between 0.9-1.1, The test is valid.

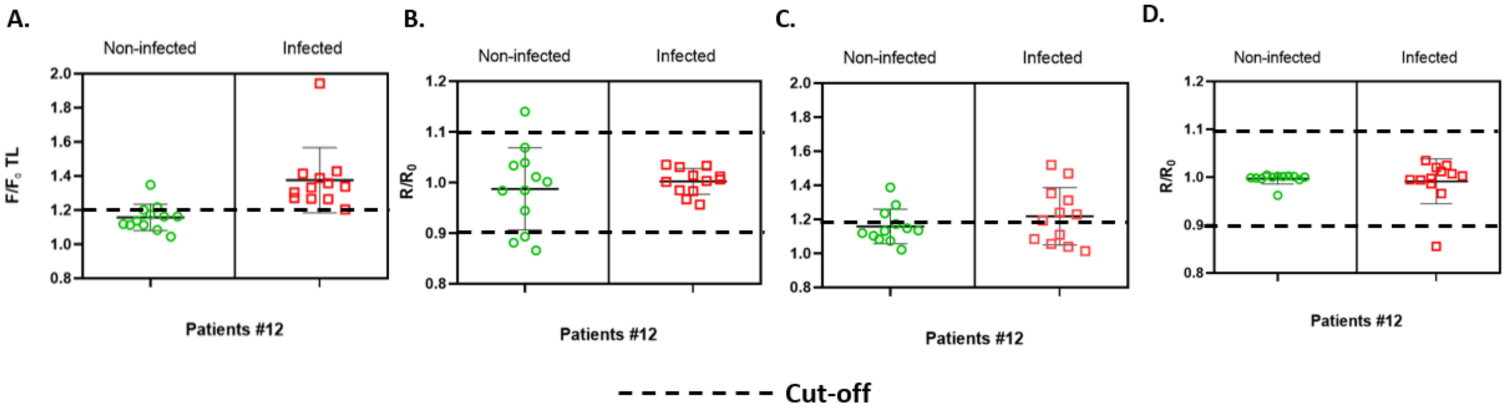

Figure S.8. Human swab samples evaluation using cut-off values. **A)** F/F_0 of E gene-gated SNPs-based LFA. **B)** R/R_0 of NSP9-gated SNPs-based LFA. **C)** F/F_0 of NSP9-gated SNPs-based LFA. **D)** R/R_0 of NSP9-gated SNPs based LFA human swab samples measurement results. According to the results, sensitivity is 100%, specificity is 75% for E gene-gated SNPs-based LFA while sensitivity is 50%, and specificity is 75% for NSP9-gated SNPs-based LFA. The numbers' reliability is insufficient; therefore, these sequences could not be optimum for CoV-2 detection.

S. Table 1. qRT-PCR confirmed infected and non-infected human swab samples NSP12-gated SNPs-based LFA F/F_0 measurement results.

**Sample Nr.**	**Non-infected F/F_0**	**Infected F/F_0**
1	1,112938	1,387454
2	1,174473	1,264701
3	1,137642	1,303458
4	1,217932	1,270121
5	1,162405	1,266209
6	1,083992	1,337972
7	1,16205	1,427794
8	1,348644	1,942938
9	1,200729	1,413668
10	1,118953	1,332821
11	1,112445	1,20363
12	1,044157	1,35711

S. Table 2. qRT-PCR confirmed infected and non-infected human swab samples E gene-gated SNPs-based LFA F/F_0 measurement results.

**Sample Nr.**	**Non-infected F/F_0**	**Infected F/F_0**
1	1,112938	1,387454
2	1,174473	1,264701
3	1,137642	1,303458
4	1,217932	1,270121
5	1,162405	1,266209
6	1,083992	1,337972
7	1,16205	1,427794
8	1,348644	1,942938
9	1,210729	1,413668
10	1,118953	1,332821
11	1,112445	1,20363
12	1,044157	1,35711

S. Table 3. qRT-PCR confirmed infected and non-infected human swab samples NSP9-gated SNPs-based LFA F/F_0 measurement results.

**Sample Nr.**	**Non-infected F/F_0**	**Infected F/F_0**
1	1,17249	1,311039
2	1,235331	1,519135
3	1,08232	1,239887
4	1,131349	1,055612
5	1,147786	1,469429
6	1,134816	1,22939
7	1,07342	1,013522
8	1,103766	1,038961
9	1,02195	1,109904
10	1,387384	1,083012
11	1,284478	1,35299
12	1,119105	1,192062
